# Quality of active case-finding for tuberculosis in India: a national level secondary data analysis

**DOI:** 10.1080/16549716.2023.2256129

**Published:** 2023-09-21

**Authors:** Hemant Deepak Shewade, G. Kiruthika, Prabhadevi Ravichandran, Swati Iyer, Aniket Chowdhury, S. Kiran Pradeep, Kathiresan Jeyashree, S. Devika, Joshua Chadwick, Jeromie Wesley Vivian, Dheeraj Tumu, Amar N. Shah, Bhavin Vadera, Venkatesh Roddawar, Sanjay K. Mattoo, Kiran Rade, Raghuram Rao, Manoj V. Murhekar

**Affiliations:** a Division of Health Systems Research, ICMR-National Institute of Epidemiology (ICMR-NIE), Chennai, India; b Division of Epidemiology and Biostatistics, ICMR-National Institute of Epidemiology (ICMR-NIE), Chennai, India; c Tuberculosis, Office of the World Health Organization (WHO) Representative to India, New Delhi, India; d School of Public Health, ICMR-National Institute of Epidemiology (ICMR-NIE), Chennai, India; e Health Office, USAID India, New Delhi, India; f TIFA project, John Snow India Private Ltd, New Delhi, India; g Central TB Division, Ministry of Health and Family Welfare, New Delhi, India

**Keywords:** Operational research, TB ACF cycle, number needed to screen, TB ACF quality indicators, high-risk groups, India

## Abstract

**Background:**

India has been implementing active case-finding (ACF) for TB among marginalised and vulnerable (high-risk) populations since 2017. The effectiveness of ACF cycle(s) is dependent on the use of appropriate screening and diagnostic tools and meeting quality indicators.

**Objectives:**

To determine the number of ACF cycles implemented in 2021 at national, state (*n* = 36) and district (*n* = 768) level and quality indicators for the first ACF cycle.

**Methods:**

In this descriptive study, aggregate TB program data for each ACF activity that was extracted was further aggregated against each ACF cycle at the district level in 2021. One ACF cycle was the period identified to cover all the high-risk populations in the district. Three TB ACF quality indicators were calculated: percentage population screened (≥10%), percentage tested among screened (≥4.8%) and percentage diagnosed among tested (≥5%). We also calculated the number needed to screen (NNS) for diagnosing one person with TB (≤1538).

**Results:**

Of 768 TB districts, ACF data for 111 were not available. Of the remaining 657 districts, 642 (98%) implemented one, and 15 implemented two to three ACF cycles. None of the districts or states met all three TB ACF quality indicators’ cut-offs. At the national level, for the first ACF cycle, 9.3% of the population were screened, 1% of the screened were tested and 3.7% of the tested were diagnosed. The NNS was 2824: acceptable (≤1538) in institutional facilities and poor for population-based groups. Data were not consistently available to calculate the percentage of i) high-risk population covered, ii) presumptive TB among screened and iii) tested among presumptive.

**Conclusion:**

In 2021, India implemented one ACF cycle with sub-optimal ACF quality indicators. Reducing the losses between screening and testing, improving data quality and sensitising stakeholders regarding the importance of meeting all ACF quality indicators are recommended.

## Introduction

Tuberculosis (TB) is the 13th leading cause of death and the second leading infectious killer after COVID-19 [1]. Globally, in 2021, there were an estimated 10.6 million people diagnosed with TB and 1.6 million TB deaths [1]. The Southeast Asia World Health Organization (WHO) region has the highest burden, with India alone accounting for 28% of incident TB and 36% of TB deaths [1].

Many people with TB are still missed: 3.9 million globally and 0.6 million in India. Active case-finding (ACF) is an option to detect missing people with TB in high-risk populations [1,2]. ACF is a systematic screening for active TB implemented outside healthcare facilities and includes household contact investigation and institutional- and community-based systematic screening of high-risk populations [3]. These high-risk populations are also called marginalised and vulnerable populations [3]. WHO recommends that ACF be done in these high-risk populations with at least 0.5% prevalence of undetected TB and not in the general population [4].

**Figure 1.**
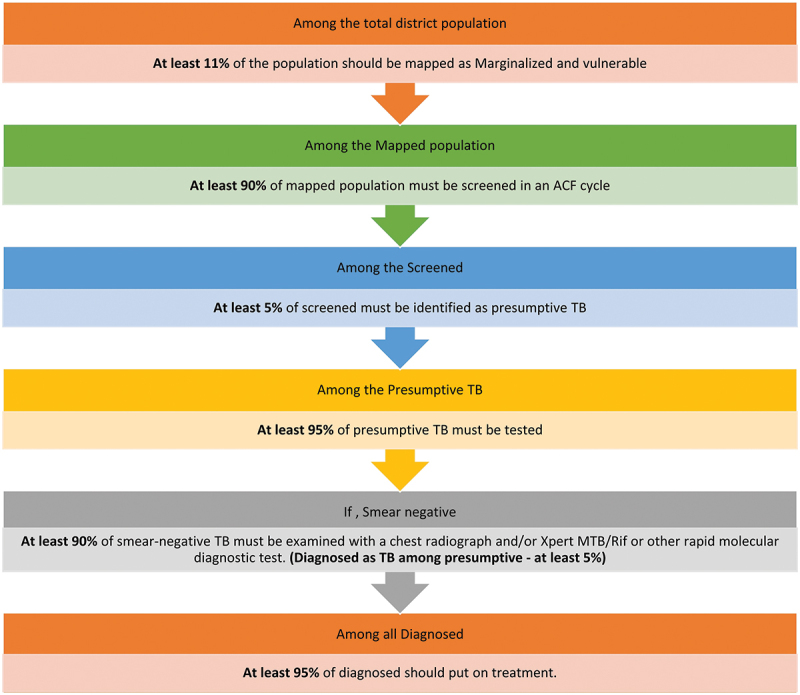
Quality indicators* for an ACF cycle for TB, India.

ACF: active case-finding; TB: tuberculosis; *these quality indicators were revised to three indicators for the purpose of this study (for reasons and other details, please refer to statistical analysis under methods).

In India, the Global Fund-supported Project *Axshya* was implemented in high-risk populations. It is one of the few community-based TB case-finding projects to have systematically assessed the benefits of ACF at the individual and community levels when compared to PCF [2,5–7]. Since 2017, India’s National TB Elimination Programme (NTEP) has been implementing ACF among marginalised and vulnerable populations in all districts [8]. The ACF quality indicators and their respective cut-offs have been identified (Figure 1) [9].

Estimated TB deaths increased in India in 2020 for the first time in a decade [10]. The death rate due to all forms of TB between 2019 and 2020 increased by 11% [10]. Compared to 2019, the case notifications dropped by 25% in 2020 [11]. The gains made have been reversed due to COVID-19, and this is a potential setback to India’s ambitious plan to attain the sustainable development goals (SDG) TB targets in 2025, 5 years ahead of the global 2030 deadline [8]. While emerging from the COVID-19 response, intensive community engagement, maintaining awareness of the importance of TB services, and ramped-up ACF are required to compensate for the missed diagnoses during the lockdown period [12].

ACF’s impact on TB transmission will depend on the scale, frequency, quality and choice of high-risk populations [13]. There is limited evidence on the frequency of ACF cycle in marginalised and vulnerable populations [4]. A trial among South African gold miners investigated the optimal frequency of TB ACF using radiological screening [14]. Compared to annual radiological screening, six monthly radiological screenings did not detect more TB, but identified clinically less extensive disease and lower deaths during treatment [14]. Two ACF cycles in a year where high-risk populations are screened twice with acceptable ACF quality indicators appear to have additional benefits over one cycle.

India NTEP commissioned this study to guide evidence-based strategic planning. This operational research was aimed at determining (at the national, state and district level) the number of ACF cycles implemented in 2021. We also measured the ACF quality indicators and the number needed to screen (NNS) for the first ACF cycle. At the state level, we also determined the correlation between i) TB prevalence and percentage diagnosed among tested, ii) TB prevalence and NNS, iii) annual presumptive TB examination rate and percentage diagnosed among tested and iv) annual presumptive TB examination rate and NNS. Finally, at national level, we calculated NNS stratified by high-risk groups.

## Methods

### Study design

A descriptive ecological design using secondary aggregate data was used in this study.

### Setting

In 2021, India had 36 state-level TB cells and 768 NTEP districts. Pre-COVID-19, India notified ≈2.4 million TB patients in 2019, which decreased to ≈1.6 million in 2020, ≈1.9 million in 2021 and finally reached ≈2.4 million in 2022 [1,15–17]. The dip in 2020–21 was due to the COVID-19 pandemic and its associated response. In 2020, ACF guidance was issued as a part of the COVID-19 mitigation guidelines. During April–June 2021, the second COVID-19 wave hit the country and starting July 2021, there was a push to implement ACF to detect the missing TB cases.

One ACF cycle is the dedicated period identified by the district when ACF will be done with the plan to cover all the district’s high-risk populations. Routinely, within a district, all high-risk populations are to be identified and added to Ni-kshay (TB information management system under NTEP). This process is called mapping. The mapped population’s details are captured against each unique mapping identifier. Against each mapped population, ACF activity-linked aggregate numbers are captured.

As per guidelines, starting in 2017, ACF cycles were to be conducted thrice a year [9]. Conducting ACF cycles over a few weeks and repeating them thrice a year required the whole health system’s support and was not possible to implement merely by the district NTEP staff. The budget for these cycles, including honorariums, had to be planned and accounted for in the district programme implementation plan prepared by the end of each financial year. In the following years, based on feasibility, many states divided each ACF cycle into multiple ACF rounds, and some even implemented ACF on fixed days a month throughout the year. Keeping resource constraints in mind, some states also decided to focus only on specific high-risk groups (not all). Hence, there were state-specific variations.

### Study population

In this national-level study, our study unit was an ACF cycle conducted in a district (*n* = 768) in 2021.

### Data extraction, variables and source of data

From Ni-kshay, aggregate TB programme data for each ACF activity in 2021 was extracted. This was further aggregated against each ACF cycle at district level.

We extracted district-level aggregate data like name of the district, name of state, ACF cycle number in the year (derived), district population, high-risk population mapped, population screened, population of presumptive TB tested and diagnosed as TB. The above data were available against multiple ACF activities or rounds, and these were linked to ACF cycles in the district. To enable this, we contacted all the states and districts and obtained the number of ACF cycles in 2021 and the date range for each ACF cycle (not available in Ni-kshay). During this process, we did not come to know of any other ACF algorithm that was systematically followed at state level other than symptom-screen (among high-risk populations) followed by sputum microscopy or Xpert MTB/Rif among symptom-screen positive (ACF-detected presumptive TB).

For state-level TB prevalence, data from the national TB prevalence survey (2019–21) were used [18]. State-level annual presumptive TB examination rate was extracted for the year 2021 from the India TB report 2022 [10].

At the national level, ACF data were also aggregated across high-risk populations: type of high-risk population, population screened, population of presumptive TB tested and diagnosed as TB.

### Statistical analysis

Data management and analysis was done using Excel (Microsoft Redmond WA, USA) and maps were made using QGIS (version 3.24.2).

While contacting the districts for clarity on ACF cycle data, we realised that mapping was not done comprehensively. There were many instances when only those areas that were planned to be screened during an ACF activity or round were identified and mapped for high-risk populations. The number of presumptive TB was not consistently captured against each ACF activity in Ni-kshay. Hence, the five ACF quality indicators (Figure 1) [9] have been derived into three as follows:

- Percentage screened among district’s population – **at least 10%** (at least 11% of district population to be mapped as high-risk * at least 90% of the mapped population should be screened = 10%).
- Percentage tested among screened – **at least 4.75%** (at least 5% of the screened to be identified as presumptive TB * at least 95% of presumptive should be tested = 4.75%).
- Percentage diagnosed (microbiologically/clinically) among tested – at least **5%** (no change).

NNS (number screened divided by number diagnosed) was also calculated. It is the number of persons who must undergo screening to diagnose one person with active TB [4]. If NNS is low, it means the quality of ACF among the screened population is good.

Assuming a minimum of 0.5% undetected TB in high-risk populations [4], and an expected yield of 13% for ACF involving symptom screen followed by sputum microscopy (50% loss between presumptive and testing) [19], the NNS would be 1538. In the same population, with an expected yield of 26% for ACF involving symptom screen followed by Xpert MTB/Rif (50% loss between presumptive and testing) [19], the NNS would be 769. Hence, conservatively, NNS ≤1538 indicated acceptable quality, ≤769 was considered good, and more than 1538 was poor. Here, the yield of TB (13–26% depending on the type of diagnostic test) is defined as the number of ACF-detected TB divided by the number of estimated undetected TB in the high-risk population that was screened.

Using the available data, the following two criteria may be used to determine if the country, state, or district is doing well in an ACF cycle: i) at least 10% of the population gets screened, at least 4.75% screened get tested and at least 5% tested get diagnosed; or ii) at least 10% of the population gets screened and NNS ≤ 1538.

The Pearson correlation coefficient (r) was used to measure the linear correlation between percentage diagnosed among tested/NNS with presumptive TB examination rate/TB prevalence.

## Results

### Frequency of ACF

All districts for whom ACF data were available implemented one ACF cycle, except for 10 districts that implemented two cycles and 5 districts that implemented three cycles. 2.9% (*n* = 41 306) of the 1.44 million TB notified from the public sector were reported as ACF-detected (see S1 and S2 Annex).

### ACF quality indicators: national and state level

At the national level, 9.3% of the population got screened, 1% screened got tested, and 3.7% of those tested got diagnosed with TB. The NNS was 2824.

**Figure 2.**
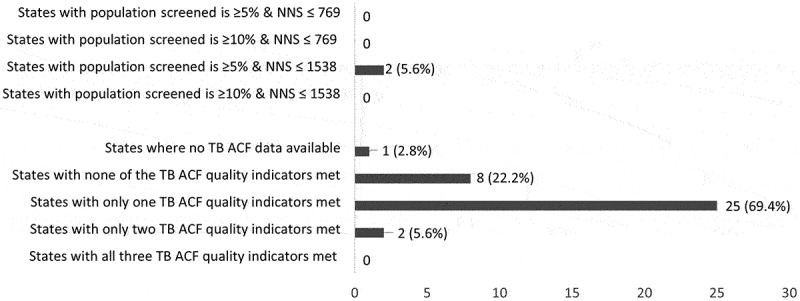
Number of states that met the TB ACF quality indicators and number needed to screen (NNS) cut-offs for TB ACF cycle (first cycle)* in 2021, India (*N* = 36).

TB: tuberculosis; ACF: active case-finding; *all states implemented one ACF cycle in 2021. There were exceptions in a few districts of Haryana, Andaman & Nicobar, Diu, Gujarat, and Uttar Pradesh. Data of the first cycle are been presented; **ACF data were not available for 111 districts. Their district population was not aggregated at state and national levels as it would underestimate the ‘percentage population screened’ indicator.

**Figure 3.**
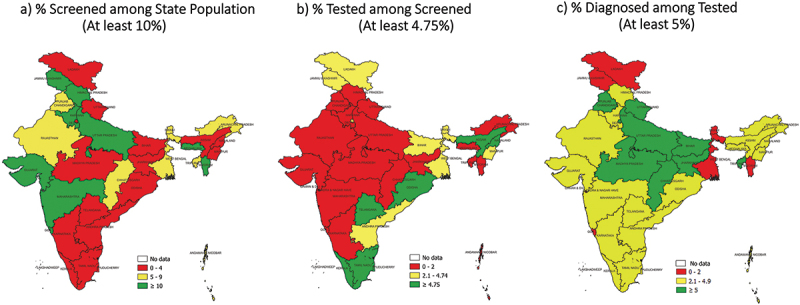
State map of India depicting the three TB ACF quality indicators (first cycle)*, 2021.

ACF: active case-finding; TB: tuberculosis; *with exception in few districts, all states implemented one ACF cycle in 2021. Data of the first cycle are presented. **ACF data were not available for 111 districts. Their district population was not aggregated at state and national levels as it would underestimate the ‘percentage population screened’ indicator.

**Figure 4.**
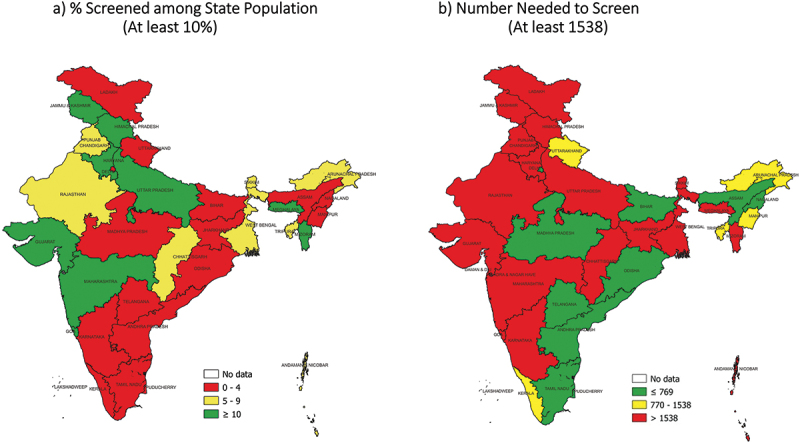
State map of India depicting the percentage population screened and number needed to screen (NNS) indicators during TB ACF cycle (first cycle)*, 2021.

ACF: active case-finding; TB: tuberculosis; *with exception in few districts, all states implemented one ACF cycle in 2021. Data of the first cycle are presented. **ACF data were not available for 111 districts. Their district population was not aggregated at state and national levels as it would underestimate the ‘percentage population screened’ indicator.

Of 36 states, 10 screened at least 10% of their population, 8 tested at least 4.75% of screened and 10 diagnosed at least 5% TB among those tested. Sixteen states met the NNS cut-off ≤1538 (see Table 1). Despite this, none of the states met all the three TB ACF quality indicators’ cut-offs. None of the states met the percentage population screened (≥10%) as well as NNS (≤1538) cut-offs (see Table 1, Figures 2–4, S3 and S4 Annex). The reason for this is as follows. Of the 16 states that met the NNS (≤1538) cut-off (yellow/green in Figure 4), 14 screened <5% of their population (red in Figure 4). Of the 10 states that met the percentage population screened (≥10%) cut-off (green in Figure 4), none of them met the NNS (≤1538) cut-off (yellow/green in Figure 4). Of the 12 states that tested, less than 1.0% of those screened; seven screened more than 10% of their population (see Table 1). Arunachal Pradesh and Tripura achieved the NNS cut-off (≤1538) and screened ≥5% of the population (but <10%) (see Figure 4).

**Table 1.** State-wise TB ACF quality indicators derived for TB ACF cycle (first cycle)* in 2021, India.

State	% Screened among population** (at least 10%)	% Tested among screened (at least 4.75%)	% Diagnosed among tested (at least 5%)	NNS (max. 1538)
**INDIA**	**9.3**	**1.0**	**3.7**	**2824**
Andaman & Nicobar	9.2	2.1	2.7	1805
Andhra Pradesh	3.5	4.2	3.3	728
Arunachal Pradesh	6.7	1.5	4.7	1455
Assam	0.4	7.4	3.2	421
Bihar	1.1	2.5	8.8	464
Chandigarh	0.5	11.5	6.7	130
Chhattisgarh	8.1	0.3	7.2	4687
Dadra Nagar Haveli/Diu Daman	45.2	0.1	2.1	33284
Delhi	0.6	2.5	19.0	213
Goa	14.8	0.6	1.6	10755
Gujarat	35.0	0.2	4.3	9545
Haryana	14.2	0.5	3.1	6091
Himachal Pradesh	18.0	0.3	2.8	11695
Jammu & Kashmir	15.1	3.4	0.9	3303
Jharkhand	1.0	0.4	18.3	1547
Kerala	0.7	5.7	2.2	816
Karnataka	2.0	0.6	3.0	5840
Ladakh	1.9	2.8	0.6	6481
Lakshadweep	1.4	52.7	0.2	957
Madhya Pradesh	4.2	1.8	7.2	747
Maharashtra	12.8	1.3	2.5	2985
Manipur	4.0	3.9	2.3	1096
Meghalaya	19.3	0.7	2.6	5814
Mizoram	18.4	1.5	1.0	6746
Nagaland	1.8	6.1	3.2	519
Odisha	0.5	9.2	2.2	485
Punjab	6.0	0.2	8.8	5961
Rajasthan	9.3	0.6	3.6	4421
Sikkim	4.7	3.6	1.1	2598
Tamil Nadu	1.1	5.3	4.2	447
Telangana	1.4	10.0	3.9	258
Tripura	6.3	1.2	8.6	953
Uttar Pradesh	17.7	0.4	6.5	3545
Uttarakhand	2.8	1.4	6.9	1066
West Bengal	7.2	2.3	1.1	4027
Puducherry	No ACF Data Available			

TB: tuberculosis; ACF: active case-finding; NNS: number needed to screen; *all states implemented one ACF cycle in 2021. There were exceptions in few districts of Haryana, Andaman & Nicobar, Diu, Gujarat, and Uttar Pradesh. Data of the first cycle are presented; **ACF data were not available for 111 districts. Their district population was not aggregated at state and national levels as it would underestimate the ‘percentage population screened’ indicator.

There was a positive correlation between the percentage diagnosed among tested and TB prevalence at the state level (r = 0.49, p-value: 0.007) (see S5 Annex). However, there was no significant correlation between TB prevalence and NNS (*r*= −0.08, p-value: 0.683) (see S6 Annex). For states with high presumptive TB examination rates, the percentage diagnosed among tested was low (*r*= −0.40, p-value: 0.016) and NNS was high (r = 0.38, p-value: 0.025) (see S7 and S8 Annex).

### ACF quality indicators: district level

Of 768 districts, in 111 (14.5%) districts, ACF data were not available (see S3 Annex). None of the districts met all three TB ACF quality indicators’ cut-offs. Thirteen districts met the percentage population screened indicator (≥10%), as well as NNS (≤1538) cut-offs. Thirty-six districts met NNS cut-off and screened ≥5% of the population (but <10%) (see S9–S12 Annex).

### National level NNS: by high-risk population type

The proportion of screened that were tested was consistently high (≥4.75%) in institutional facilities and very low (≤1%) for population-based groups. Correspondingly, for the following groups, the NNS was ≤769: immunosuppressive treatment, elderly, homeless and night shelters, old age homes and orphanages, prisons, and street children. For the following groups, the NNS was 770–1538: people with cardiovascular diseases, diabetes mellitus, malnutrition-prone areas, asylum, refugee camps and people consuming raw milk and uncooked meat. NNS was >1538 for most population-based high-risk groups (see Table 2).

**Table 2.** TB ACF quality indicators derived for each high-risk population during TB ACF cycle (first cycle)* in 2021, India.

High-risk population groups	Number screened	Number tested	% Tested	Number diagnosed	% Diagnosed	NNS
**Clinical**						
Asthma & COPD	30232	636	2.1	16	2.5	1890
COVID recovered	616088	9209	1.5	174	1.9	3541
CVDs	19603	119	0.6	21	17.6	933
DM and HTN	448106	11496	2.6	356	3.1	1259
Immunosuppressives	3484	336	9.6	11	3.3	317
Renal & Liver disease	47319	131	0.3	0	0.0	0
Cancer	1302	12	0.9	0	0.0	0
Palliative Care	523	114	21.8	0	0.0	0
High malnutrition	1245564	16100	1.3	1063	6.6	1172
**Socio-economic**						
Elderly >60 years	128638	3581	2.8	209	5.8	615
Children <10 years	104757	127	0.1	4	3.1	26189
Occupation*	1366444	25012	1.8	815	3.3	1677
Unorganised labour	2455829	49045	2.0	1170	2.4	2099
Homeless &Night shelters	46862	2582	5.5	89	3.4	527
NACO/SACS identified HRG for HIV	883885	9076	1.0	403	4.4	2193
Old age homes & Orphanages	42810	1878	4.4	113	6.0	379
Prisons inmates	117665	6358	5.4	164	2.6	717
Asylums & Refugee camps	36465	1007	2.8	41	4.1	889
Slum	18321022	146781	0.8	5118	3.5	3580
Street children	429	86	20.0	4	4.7	107
**Geographic**						
Difficult to reach villages	15829483	153403	1.0	6922	4.5	2287
Population consuming raw milk and uncooked meat	330522	3593	1.1	254	7.1	1301
Tribal areas huts & hostels	3264889	14629	0.4	927	6.3	3522
Villages seeking care from traditional healers	1898795	11285	0.6	531	4.7	3576
Villages with high case load	877331	12899	1.5	546	4.2	1607
**Unknown**						
Others	68780092	436000	0.6	17737	4.1	3878

TB-tuberculosis; ACF- active case-finding; NNS- number needed to screen; COPD- chronic obstructive pulmonary disease; DM – Diabetes Mellitus; HTN – Hypertension; CVD- cardiovascular diseases; NACO- National AIDS control organization; SACS- State AIDS control society; HRG- High risk group; HIV- Human immunodeficiency virus; *occupation includes construction site workers, cotton mill workers, Mine workers, stone crushers, Tea Garden workers, weaving glass industrial workers.

## Discussion

### Summary of findings

This is the first national-level assessment of the scale, frequency and quality of ACF for TB in India. In 2021, most states implemented one ACF cycle (aimed to cover the high-risk populations once in a year) with sub-optimal ACF quality indicators. States that reported high coverage of population had sub-optimal indicators of testing and diagnosis among the screened population. States that reported high levels of testing and diagnosis among the screened population had sub-optimal indicators pertaining to coverage. The percentage diagnosed among tested was high, and NNS was low in states with high TB prevalence and/or low presumptive TB examination. The NNS was consistently low (acceptable) for ACF in institutional facilities and high (above the 1538 cut-off) for population-based groups.

### Limitations of the study

There were a few limitations, mostly due to the data available in Ni-kshay. As discussed before, we were not able to assess all ACF quality indicators (Figure 1). Specifically, we could not calculate the percentage population that was mapped, the percentage of the mapped population that was screened, the percentage of presumptive identified among the screened population and the percentage of testing among presumptive TB.

In 2021, comprehensive mapping of the high-risk populations in the district was not done at the beginning of the year. The COVID-19 pandemic possibly was the reason for this. Mapping, if done, was done in the high-risk populations that were targeted in specific ACF rounds conducted post-June 2021. The derived indicator, the percentage of the population screened, even if ≥10%, does not guarantee that >90% of high-risk populations were covered.

Our current assessment of ACF was based on the NTEP guidelines, for which the indicators were initially developed only at the national level. While the output of ACF would depend on background respiratory conditions prevalent in a given geography, the TB prevalence and the type of test used, single national benchmarking for comparing states and districts across the country is a limitation of our current assessment. However, this was the best possible way in the current situation. Based on prediction models, differential benchmarking will be done in the future.

### Comparison with previous studies

The percentage tested that were diagnosed contrasts with the results of Project *Axshya* in 2013–14, which initiated ACF in 300 districts of India, where nearly 8% of those tested were diagnosed [20]. The proportion of states meeting the 5% cut-off for the percentage diagnosed among tested (28%) and high NNS (>1538) in 2021 is similar to the 2018–19 pre-COVID-19 years [21]. Low NNS for people in institutional facilities and high NNS for population-based groups were similar to the finding from a systematic review and meta-analysis (2010–20) from India. Household contact investigation and facility-based screening, especially in hospital settings, had low-weighted NNS estimates, among others [22].

### Implications for ACF in India

The possible reasons and recommendations based on our findings are as follows. These require extensive sensitisation up to district and state levels, and those relevant for a state/district may be taken up and acted upon. These points may also be considered while revising the existing national ACF guidance document [9].

First and foremost, there is a need to sensitise what one ACF cycle means, how many cycles are required per year with good-quality indicators to impact TB epidemiology and the realistic expectation (yield) from an ACF cycle or ACF round/activity within a cycle. The need to take corrective steps based on frequent quality check mechanisms involving all ACF quality indicators (not merely the proportion diagnosed among tested), NNS and yield (extent of undetected TB in the ACF-detected population) must be reinforced. Before this is done, districts should clearly understand the date range between which the ACF activities will constitute one ACF cycle. Upon contacting the districts and states to understand the number of ACF cycles and date range for each, only a few had clarity and relevant information.

The NTEP should also revise its guidance and change the recommendations from three ACF cycles per year (January/July/December) to two ACF cycles per year. Doing back-to-back ACF in two consecutive months (December and January) may not be recommended. Covering the adequately mapped high-risk groups at least twice a year (at least once a year in resource-constrained settings) with acceptable ACF quality indicators should be expected from districts. Also, in resource-constrained settings, covering the same high-risk population within 6 months is not advisable, which was also anecdotally observed.

In resource-constrained settings, some districts and states may also consider two ACF cycles a year in select high-risk groups (not all). In other words, they may implement two ACF cycles a year, with, say, 40-50% coverage of high-risk populations in the district, and the other ACF quality indicators should be acceptable (see Figure 1). NTEP may begin with easily identifiable high-risk target groups and then widen their scope of activities [23].

Second, comprehensive mapping of the whole of the district at the beginning of the year may not be happening, which may result in possibly choosing low-burden populations (ACF is not recommended in populations with <0.5% prevalence of undetected TB) [4] and missing cases due to inadequate coverage of high-risk population.

Third, the percentage screened population that were tested fared the worst among the three TB ACF quality indicators. This indicator was even poorer for population-based screening and difficult to reach population groups. This finding could be because either the operational definition of presumptive TB was not correctly followed, or systematic mechanisms of specimen transfer were not in place. We speculate that this may also be due to inadequate planning and budgeting (including honorariums for volunteers for specimen collection and transport). In other words, ACF-detected presumptive TB patients may be expected to visit the nearest diagnosing facility on their own, resulting in attrition.

Fourth, the possible use of less sensitive diagnostic tools like sputum microscopy may be one of the reasons why the cut-off for the percentage diagnosed among tested was not met. Another reason could be poor specimen quality in ACF settings.

Fifth, the mechanisms to randomly cross-check the aggregate numbers reported from an ACF activity in Ni-kshay (number screened, number presumptive TB, number tested and number ACF-detected TB patients) appear inadequate (anecdotal observation). The ACF guidance mentions using observers nominated by the state to assess the ACF quality (but not about specific mechanisms to cross-check or verify the aggregate numbers reported from an ACF cycle/activity) [9]. Some 'reported' ACF-detected patients may not be truly ACF-detected. They may be PCF-detected in true sense. In other words, not all patients detected during the period of ACF activity may be detected as a result of ACF. Focusing only on the indicator ‘proportion diagnosed among tested’ and ignoring other ACF quality indicators could be one of the reasons for this misclassification (anecdotal observation).

Most of the states that reported a high percentage of population screened, with exceptions few and far between, fared poorly in NNS. This makes screening of such large populations (even if true) futile. With systematic individual-level paper-based data capture starting from those screened on the ground (‘field activity daily report form’ as per 2017 ACF guidance) [24], it is possible to cross-check these numbers. In the updated 2019 ACF guidance developed by the state of Haryana, the 'field activity daily report form' did not find a mention [9]. If any revisions are made in the ACF guidance in the future, the ‘field activity daily report form’ should be incorporated.

Sixth, data should be captured in Ni-kshay, ACF cycle-wise, with a prior understanding of the ACF cycle date range. At the district level, this may be simplified and involve the following district-level aggregate numbers: total district population, total high-risk population, total high-risk population by risk group, within each risk group, the numbers screened, presumptive, tested and diagnosed. Monitoring mechanisms should be implemented to ensure that these are aggregated from the TU level along with specified mechanisms to randomly cross-check these numbers (referring to paper-based data used in the field during ACF).

Finally, adequate planning may not be done in advance, and therefore, adequate budgets may not be requested in programme implementation plans, resulting in inadequate funds to implement ACF cycles.

These findings and recommendations require further systematic qualitative exploration, which is underway (March–June 2023). A better granular understanding of the ACF care cascade is required to generate all five ACF quality indicators, predictors and the extent of use of rapid molecular tests. For this, prospective data collection is underway in 30 randomly sampled study districts (January–December 2023).

## Conclusion

The impact of ACF for TB among high-risk populations will depend on the scale, frequency and quality of ACF. For the year 2021, we assessed this at national, state and district levels in India. India is far away from implementing at least two ACF cycles per year (i.e. covering the high-risk populations at least twice in a year) with acceptable ACF quality indicators and NNS. As India gears up to achieve the SDG 2030 TB targets, these ACF quality indicators will act as a baseline for future analyses. The recommendations based on our findings may assist in achieving the impact on TB case detection and other outcomes of ACF. This will take India closer to achieving the SDG TB target of reducing TB incidence by 80% in 2030 (when compared to 2015) [25].

## Supplementary Material

Supplemental Material

## Data Availability

Figshare: Shewade HD et al. study 2023, phase I India TB ACF Evaluation study data, https://doi.org/10.6084/m9.figshare.23723748 Data are available under the terms of the Creative Commons Attribution 4.0 International License (CC BY 4.0).

## Paper context

Active case-finding is the proactive screening of all high-risk groups for TB symptoms outside healthcare facilities. The effectiveness of active case-finding cycle(s) is dependent on the use of appropriate screening and diagnostic tools and meeting quality indicators. The frequency and quality of India’s active case-finding cycles for TB for the year 2021 was assessed. India implemented one active case-finding cycle with sub-optimal quality indicators. This requires regular sensitisation of stakeholders regarding the importance of meeting quality indicators.
