# Supplementary material for: Quality of active case-finding for tuberculosis in India: a national level secondary data analysis
Source: Glob Health Action. 2023 Sep 21;16(1):2256129. doi: 10.1080/16549716.2023.2256129 (PMC10515680; doi:10.1080/16549716.2023.2256129)
Supplement: Supplemental Material [file ZGHA_A_2256129_SM2351.docx]

**Supplementary tables and figures**

**S1 Annex: State wise total population screened, tested and diagnosed during TB ACF cycle (first cycle)* in 2021, India**

| **State** | **Total population**** | **Number screened** | **Number tested among screened** | **Number diagnosed among tested** |
| --- | --- | --- | --- | --- |
| **INDIA** | **1253043766** | **116649087** | **1110301** | **41306** |
| Andaman & Nicobar | 390690 | 36104 | 753 | 20 |
| Andhra Pradesh | 52887016 | 1862856 | 77960 | 2560 |
| Arunachal Pradesh | 1667277 | 112064 | 1633 | 77 |
| Assam | 35475942 | 148890 | 11038 | 354 |
| Bihar | 116284977 | 1291776 | 31691 | 2782 |
| Chandigarh | 1186275 | 5968 | 684 | 46 |
| Chhattisgarh | 29579112 | 2385625 | 7067 | 509 |
| Dadra Nagar Haveli /Diu Daman | 73628 | 33284 | 47 | 1 |
| Delhi | 14768333 | 94911 | 2344 | 445 |
| Goa | 870632 | 129059 | 760 | 12 |
| Gujarat | 70805903 | 24768417 | 60350 | 2595 |
| Haryana | 29906244 | 4245206 | 22713 | 697 |
| Himachal Pradesh | 2601904 | 467800 | 1450 | 40 |
| Jammu & Kashmir | 15234179 | 2305657 | 78733 | 698 |
| Jharkhand | 11295980 | 112907 | 398 | 73 |
| Kerala | 34559927 | 250433 | 14249 | 307 |
| Karnataka | 52637107 | 1068693 | 6070 | 183 |
| Ladakh | 349636 | 6481 | 180 | 1 |
| Lakshadweep | 66480 | 957 | 504 | 1 |
| Madhya Pradesh | 85670503 | 3597418 | 66488 | 4817 |
| Maharashtra | 127228338 | 16246964 | 217681 | 5443 |
| Manipur | 3168531 | 128199 | 5003 | 117 |
| Meghalaya | 3737760 | 720901 | 4686 | 124 |
| Mizoram | 1280292 | 236108 | 3602 | 35 |
| Nagaland | 1816817 | 33235 | 2025 | 64 |
| Odisha | 9566816 | 49486 | 4564 | 102 |
| Punjab | 16303082 | 971570 | 1852 | 163 |
| Rajasthan | 79945990 | 7404754 | 47042 | 1675 |
| Sikkim | 667721 | 31176 | 1107 | 12 |
| Tamil Nadu | 82430313 | 909603 | 48564 | 2033 |
| Telangana | 38191252 | 531277 | 53118 | 2058 |
| Tripura | 3988666 | 249625 | 3058 | 262 |
| Uttar Pradesh | 220006486 | 38917120 | 168335 | 10979 |
| Uttarakhand | 10849621 | 303745 | 4136 | 285 |
| West Bengal | 97550337 | 6990818 | 160416 | 1736 |
| Puducherry | No ACF data available | | | |

TB-tuberculosis; ACF- active case finding; *All states implemented one ACF cycle in 2021. There were exceptions in few districts of Haryana, Andaman & Nicobar, Diu, Gujarat, and Uttar Pradesh. Data of first cycle has been presented; ** ACF data was not available for 111 districts. Their district population was not aggregated at state and national level as it would underestimate the ‘percentage population screened’ indicator

**S2 Annex: State wise total public notified TB cases and the TB ACF-detected cases in 2021, India.**

| **State** | **Public notified TB**  **(from 2022 India TB report)** | **TB ACF cases diagnosed among tested (reported as ACF-detected)** | **%** |
| --- | --- | --- | --- |
| **INDIA** | **1443305** | **41659** | **2.9** |
| Andaman & Nicobar | 502 | 20 | 4.0 |
| Andhra Pradesh | 62100 | 2560 | 4.1 |
| Arunachal Pradesh | 2710 | 77 | 2.8 |
| Assam | 29389 | 354 | 1.2 |
| Bihar | 61467 | 2782 | 4.5 |
| Chandigarh | 4220 | 46 | 1.1 |
| Chhattisgarh | 23644 | 509 | 2.2 |
| Dadra Nagar Haveli/ Diu Daman | 948 | 1 | 0.1 |
| Delhi | 67726 | 445 | 0.7 |
| Goa | 1648 | 12 | 0.7 |
| Gujarat | 92888 | 2595 | 2.8 |
| Haryana | 45741 | 1050 | 2.3 |
| Himachal Pradesh | 12997 | 40 | 0.3 |
| Jammu & Kashmir | 9458 | 698 | 7.4 |
| Jharkhand | 35146 | 73 | 0.2 |
| Kerala | 15364 | 307 | 2.0 |
| Karnataka | 52922 | 183 | 0.3 |
| Ladakh | 280 | 1 | 0.4 |
| Lakshadweep | 12 | 1 | 8.3 |
| Madhya Pradesh | 110814 | 4817 | 4.3 |
| Maharashtra | 110136 | 5443 | 4.9 |
| Manipur | 1251 | 117 | 9.4 |
| Meghalaya | 3281 | 124 | 3.8 |
| Mizoram | 1480 | 35 | 2.4 |
| Nagaland | 2924 | 64 | 2.2 |
| Odisha | 45047 | 102 | 0.2 |
| Punjab | 35870 | 163 | 0.5 |
| Rajasthan | 103011 | 1675 | 1.6 |
| Sikkim | 1272 | 12 | 0.9 |
| Tamil Nadu | 64456 | 2033 | 3.2 |
| Telangana | 41484 | 2058 | 5.0 |
| Tripura | 2433 | 262 | 10.8 |
| Uttar Pradesh | 314042 | 10979 | 3.5 |
| Uttarakhand | 17290 | 285 | 1.6 |
| West Bengal | 69352 | 1736 | 2.5 |
| Puducherry | No ACF data available | | |

TB-tuberculosis; ACF- active case finding

**S3 Annex: List of districts where TB ACF cycle data was not available, India, 2021**

| **S. No** | **State** | **Districts with no ACF data** |
| --- | --- | --- |
| **1** | Arunachal Pradesh | Namsai |
| 2 | Bihar | Arwal, Auranangabad–BI, Banka, Krishnaharj, Saharsa |
| 3 | Chhattisgarh | Bijapur, Kondagaon |
| 4 | Dadra Nagar Haveli/ Diu Daman | Daman, Dadra & Nagar Haveli |
| 5 | Delhi | BSA chest clinic, GTB chest Clinic, LN chest clinic, LRS, Moti Nagar, SGM chest clinic |
| 6 | Himachal Pradesh | Chamba, Kangra, Kinnaur, Kullu, Lahul & spiti, Shimla, Sirmaur, Solan |
| 7 | Jharkhand | Bokaro, Chatra, Deoghar, Dhanbad, Garhwa, Giridih, Gumla, Hazaribagh, Jamtara, Khunti, Latehar, Lohardaga, Pakur, PaschimiSinghbhum, PurbiSinghbhum, Ramgarh, Sahibganj, Simdega |
| 8 | Karnataka | Bagalkot, Banglore rural, Bijapur, Chickmangalur, Davangare, Dharwad, Mysore, Raichur, Kolar, Koppal, Gulbarga |
| 9 | Maharashtra | Aurangabad MC, Jalna, Nagpur MC |
| 10 | Nagaland | Mon |
| 11 | Odisha | Anughul, Baleshwar, Bargarh, Bhadrak, Bhubaneshwar MC, Boudh, Deogarh, Dhenkanal, Gajapati, Ganjam, Jagatsinghapur, Jajapur, Jharsugiuda, Kandhamal, Kendrapara, Kendujhar, Khordha, Koraput, Malkangiri, Mayurbhanj, Nabarangapur, Nayagarh, Nuapada, Puri, Rayagada, Sambalpur |
| 12 | Puducherry | Puducherry |
| 13 | Punjab | Nawanshahr, Pathankot, Patiala, Rupnagar, Sangrur, Tarn Taran, Moga, Mohali, Jalandar, Kapurthala, Fatehgarh Sahib, Fazilka |
| 14 | Rajasthan | Sirohi |
| 15 | Tamil Nadu | West Chennai, East chennai |
| 16 | Uttar Pradesh | Auraiya, Baghpat, Gautam budh nagar, Kaushambi, Pratagarh, Sitapur |
| 17 | Uttarakhand | Pithoragarh, Uttarkashi |
| 18 | West Benghal | Uttar Dinajpur |

TB-tuberculosis; ACF- active case finding

**S4 Annex: List of states that met the TB ACF quality indicators and number needed to screen (NNS) cut-offs for TB ACF cycle (first) in 2021, India (N=36)**

| **1** | **States that met the TB ACF quality Indicators** |
| --- | --- |
|  | **List of States with all three TB ACF quality indicators met.**  Nil |
|  | **List of States with only two TB ACF quality indicators met.**  Uttar Pradesh and Chandigarh |
|  | **List of States with only one TB ACF quality indicators met.**  Andaman & Nicobar, Dadra Nager Haveli Diu Daman, Goa, Gujarat, Haryana, Himachal Pradesh, Jammu & Kashmir, Maharashtra, Meghalaya, Mizoram, Assam, Kerala, Lakshadweep, Nagaland, Odisha, Tamil Nadu, Telangana, Bihar, Chhattisgarh, Delhi, Jharkhand, Madhya Pradesh, Punjab, Tripura, Uttarakhand |
|  | **List of States with none of the TB ACF quality indicators met.**  Andhra Pradesh, Arunachal Pradesh, Karnataka, Ladakh, Manipur, Rajasthan, Sikkim, West Benghal |
|  | **List of States with no TB ACF data available**  Pondicherry |
| **2** | **States that met the % Screened population and NNS cut-offs** |
|  | **List of States with NNS met (</= 1538) and % Screened is >/=10%**  Nil |
|  | **List of States with NNS met (</= 1538) and % Screened is >/=5%**  Arunachal Pradesh and Tripura |
|  | **List of States with NNS met (</= 769) and % Screened is >/=10%**  Nil |
|  | **List of States with NNS met (</= 769) and % Screened is >/=5%**  Nil |

TB-tuberculosis; ACF- active case finding; NNS: number needed to screen

**S5 Annex: Figure showing correlation between state level TB prevalence (2021) and test positivity for TB ACF cycle (first) in 2021, India**


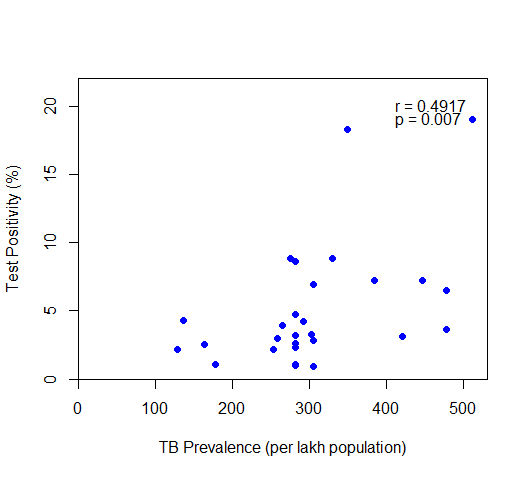


TB-tuberculosis; ACF- active case finding; r- correlation coefficient; p- p value

**S6 Annex: Figure showing correlation between state level TB prevalence (2021) and number needed to screen (NNS) for TB ACF cycle (first) in 2021, India**


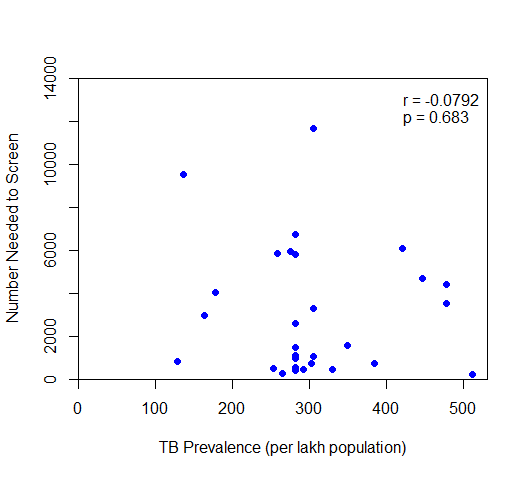


TB-tuberculosis; ACF- active case finding; NNS- number needed to screen; r- correlation coefficient; p- p value

**S7 Annex: Figure showing correlation between state level annual presumptive TB examination rate (2021) and test positivity for TB ACF cycle (first) in 2021, India**


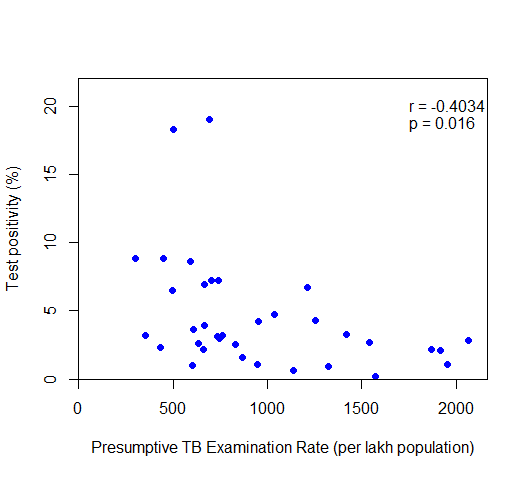


TB-tuberculosis; ACF- active case finding; r- correlation coefficient; p- p value

**S8 Annex: Figure showing correlation between state level annual presumptive TB examination rate (2021) and number needed to screen (NNS) for TB ACF cycle (first) in 2021, India**


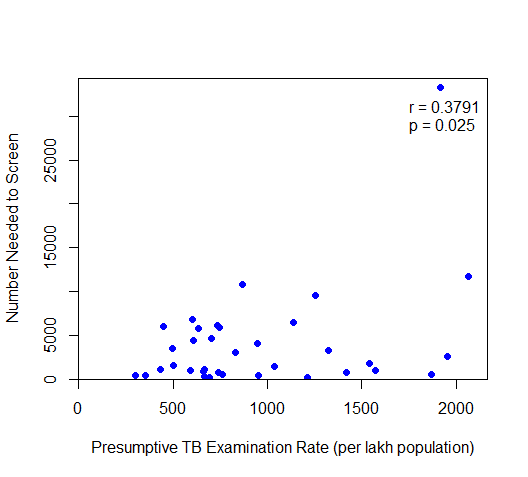


TB-tuberculosis; ACF- active case finding; NNS- number needed to screen; r- correlation coefficient; p- p value

**S9 Annex:** **Number of NTEP districts that met the TB ACF quality indicators’ and number needed to screen (NNS) cut-offs for TB ACF cycle (first cycle) * in 2021, India (N= 768)****


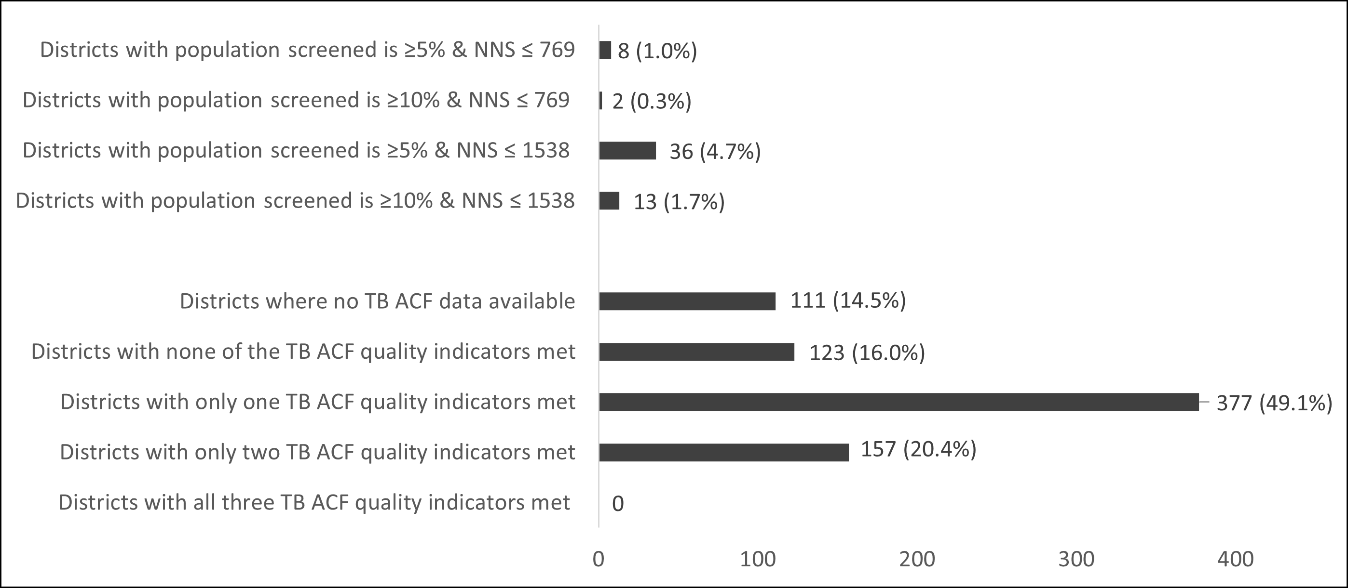


TB-tuberculosis; ACF- active case finding; *All states implemented one ACF cycle in 2021. There were exceptions in few districts of Haryana, Andaman & Nicobar, Diu, Gujarat, and Uttar Pradesh. Data of first cycle has been presented; ** ACF data was not available for 111 districts, the denominator of 768 was retained for calculation of %

**S10 Annex:** **District map of India depicting the three TB ACF quality indicators (first cycle)*, 2021**


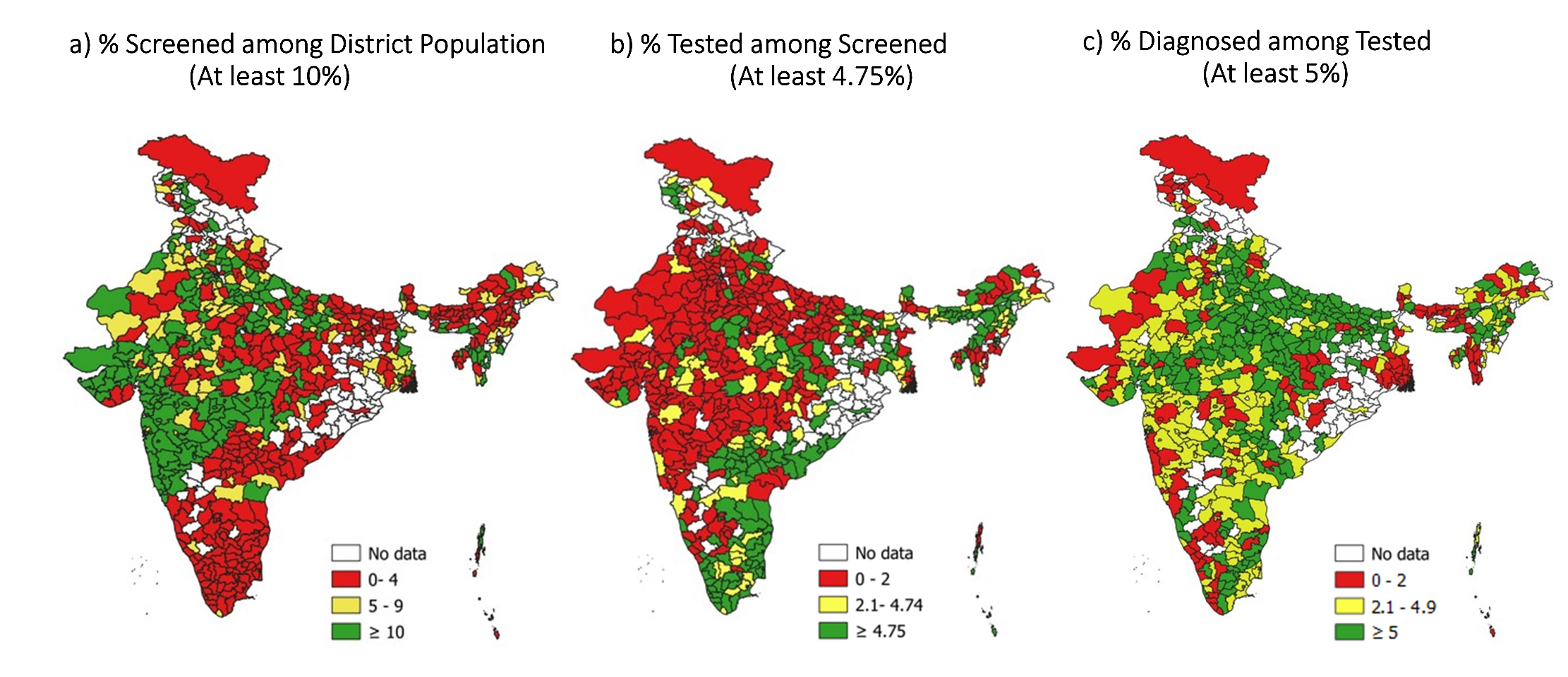


ACF: active case finding; TB: tuberculosis; *With exception in few districts, all states implemented one ACF cycle in 2021. Data of first cycle has been presented. ** ACF data was not available for 111 districts. Their district population was not aggregated at state and national level as it would underestimate the ‘percentage population screened’ indicator.

**S11 Annex:** **District map of India depicting the percentage population screened and number needed to screen (NNS) indicators during TB ACF cycle (first cycle)*, 2021**


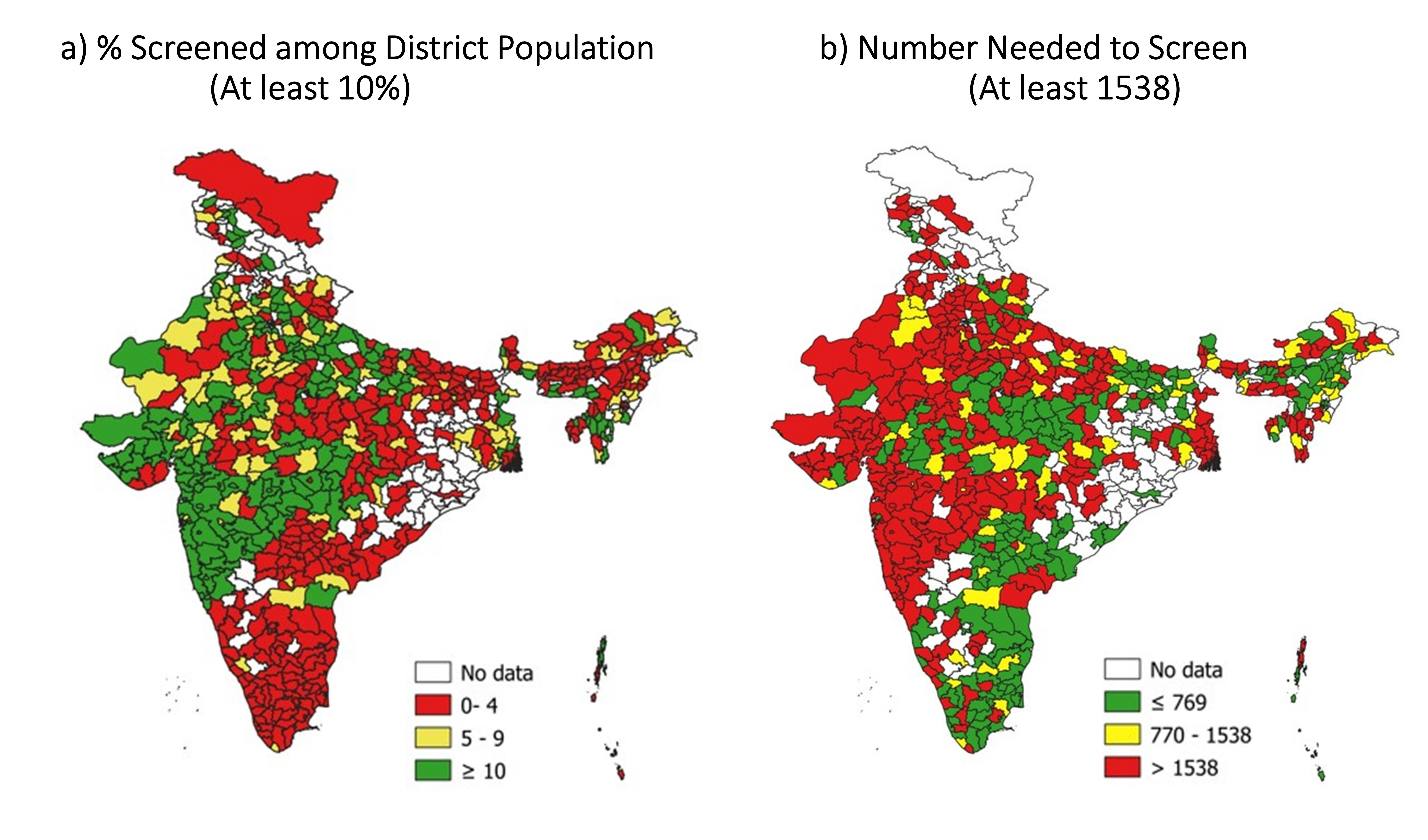


ACF: active case finding; TB: tuberculosis; *With exception in few districts, all states implemented one ACF cycle in 2021. Data of first cycle has been presented. ** ACF data was not available for 111 districts. Their district population was not aggregated at state and national level as it would underestimate the ‘percentage population screened’ indicator

**S12 Annex: List of districts that met the TB ACF quality indicators and number needed to screen (NNS) cut-offs for TB ACF cycle (first) in 2021, India (N=768)**

| **1** | **NTEP districts that met the TB ACF quality Indicators** |
| --- | --- |
|  | **List of districts with all three TB ACF quality indicators met.**  Nil |
|  | **List of districts with only two TB ACF quality indicators met.**  **Andaman & Nicobar**: South Andaman  **Andhra Pradesh:** Prakasam, Cuddapah, Vizianagaram, West Godavari  **Arunachal Pradesh**: Kurung Kumey  **Assam**: Cachar, Golaghat, Marigaon, Nagaon, North Cachar Hills  **Bihar:** Buxar, Darbhanga, Gaya, Jamui, Katihar, Khagaria, Madhepura, Muzaffarpur, Purba champaran, Saran, Sitamarhi  **Chandigarh:** Chandigarh  **Chattisgarh:** Bilaspur, Kanker, Dantewada, Raigarh  **Delhi:** Bijwasan, Jhandwalan, Karawal Nagar, Nehru Nagar, RTRM chest clinic, SPM marg  **Madhya Pradesh**: Balaghat, Harda, Hoshangabad, seoni, Shajapur, singrauli, Agar Malwa, Anuppur, Ashoknagar, Datia, Gwalior, Jabalpur, Jhabua, Katni, Satna, Shahdol, Sheopur, Shivpuri, Sidhi  **Maharashtra:** Akola MC, Byculla, Dhule MC, Gadchiroli, Hingoli, Jalgaon MC, Latur, Malegaon Corp, Nanded Waghela MC, Ulhasnagar MC  **Meghalaya**: East Khasi hills, South Garo hills  **Nagaland:** Longleng, Peren, Tuensang  **Punjab**: Barnala, Muktsar  **Tamil Nadu:** Cuddalore, Dindigul, Erode, Kancheepuram, Madurai, Perambalur, Theni, Tiruchirapalli, Tirunelveli, Villupram, Vellore  **Telangana**: Bhadradri Kothagudem, Jagtial, Jangaon, Mulugu, Narayanpet, Rajanna sircilla, Suryapet  **Tripura**: Dhalai  **Uttar Pradesh**: Agra, Aligarh, Ayodhya, Azamgarh, Bahraich, Balrampur, Bareilly, Bijnor, Chandauli, Chitrakoot, Deoria, Etah, Farrukhabad, Gonda, Hamirpur-UP, Hapur, Hardoi, Hathras, Jalaun, Jhansi, Kannauj, Kanpur Dehat, Kheri, Lalitpur, Luknow, Maharajganj, Mahoba, Mainpuri, Mau, Meerut, RaeBareli, Saharanpur, Sant Ravidas Nagar, Shahjahanpur, Shamli, Sultanpur, Ambedkar Nagar, Jyotiba phule Nagar, Mathura, Sambhal, Sant Kabir Nagar, Sonbhadra  **Uttarkhand:** Champawat, Garhwal  **West Benghal**: Bagbazar, Manshatala, Strand Bank, Tangra  **Rajasthan:** Ganganagar, Jhalawar, Pratapgarh  **Himachal Pradesh**: Bilaspur HP  **Jharkhand**-Dumka, Godda, Ranchi  **Karnataka**: Bidar, Gadag, Udupi  **Gujarat**: Ahmedabad MC, Anand, Arvalli, Bharuch, Dahod, Devbhumi Dwarka, Narmada, Sabarkantha, Surat Rural  **Kerala:** Palakkad  **Haryana:** Panipat |
|  | **List of districts with only one TB ACF quality indicators met.**  **Andaman**- North & Middle Andaman, Nicobars  **Andhra Pradesh**- Anantpur, Chittoor, East Godavari, Nellore, Srikakulam, Krishna  **Arunachal Pradesh**- West Siang, Upper Siang, West Kameng, Dibang valley, Lower Subansiri, Namsai  **Assam**- Baksa, BArpeta, Bongaigaon, Chirang, darrang, Dhemaji, Dhbri, Dibrugarh, Hailakandi, Jorhat, Kamrup, Kamrup Metro, Karbi Anglong, Karimganj, Lakhimpur, Nalbari, Sibasagar, Sonitpur  **Bihar:** Patna, Sheikhpura, Sheohar, Araria, Begusarai, Bhagalpur, Gopalganj, Jehanabad, Kaimur, Lakhisarai, Madhubani, Munger, Nalanda, Nawada, Paschim champaran. Rohtas, Samastipur, Siwan, Supaul, Vaishali  **Chattisgarh**- Durg, Mahasamund, Balrampur, Kabirdham, Korba, Koriya, Sukma, Balod, baloda Bazar, Bastar, Jashpur, Mungeli, Rajnandgaon, Sarguja  **Dadra**- Diu  **Delhi**- Shahadra, BJRM chest clinic, DDU chest clinic, Gulabi Bagh, Hedgewar chest clinic, NDMC, Patparganj, RK mission, SPMH chest clinic  **Madhya Pradesh**-Mandla, Mandsaur, Narsinghpur, Raisen, Betul, Damoh, Neemuch, Alirajpur, Barwani, Bhind, Bhopal, Bhurhanpur, Chhatarpur, Dhar, Dindori, Guna, Indore, Khargone, Morena, Panna, Rajgarh, Ratlam, Rewa, Sagar, Tikamgarh, Umaria, Vidisha  **Maharashtra-** Ahmadnagar, Akola, Amravati, Amravati MC, Andheri East, Aurangabad, Bail Bazar road, Bandra East, Bandra West, Beed, Bhandara, Borivali, Buldana, Centenary, Chandrapur, Chembur, Colaba, dadar, Dahisar, Dhule, Ghatkopar, Gondhiya, Goregaon, Govandi, Grant road, Jalgaon, Kandivali, Kohlapur, Kohlapur MC, Kurla, Malad, Mira Bhayandar, Mulund, Nagpur, Nanded, Nandurbar, Nashik, Nashik Corp, Navi Mumbai, Osmanabad, palghar, Parbhani, Parel, Pimpri chinchwad, Prabhadevi, Pune MC, Pune rural, Raigad, Ratnagiri, Sangli, Sangli MC, Satara, Sindhudurg, Sion, Solapur, Solapur MC, Thane, Thane MC, Vasai Virar, Vikhroli, Wardha, Washim, Yavatmal, Bhiwandi Nizampur  **Manipur:** Chandel, Bishnupur, Imphal East, Imphal West, Tamenglong, Thoubal, Ukhrul  **Meghalaya**: East Garo Hills, Jaintia Hills, Ribhoi  **Mizoram:** Aizwal, Champhai, Kolasib, Mamit, Saiha, Serchhip, Lunglei  **Nagaland:** Kiphire, Kohima, Mokokchung, Phek, Wokha  **Odisha:** Cuttak, Klahandi, Sonapur  **Punjab:** Firozpur  **Sikkim:** West District, DTC Gangtok, North District, Singtam  **Tamil Nadu:** Central Chennai, East Chennai, Krishnagiri, Nagapattinam, North Chennai, Ramanathapuram, Thanjavur, Nilgris, Thiruvallur, Thiruvarur, Thoothukudi, Tiruvannamalai, karur, Virudhunagar  **Telangana**- Hyderabad, Jogulamba Gadwal, Karimnagar, Khammam, Kumurambheem Asifabad, Mahabubabad, Mahabubnagar, Medak, Medcahl, Malkajgiri, Nagarkurnool, Nalgonda, Rangareddy, Sangareddy, Siddipet, Vikarabad, Yadadri Bhuvanagiri, Adilabad, Jayashankar Bhupalpally, Mancherial, Nirmal, Peddapalli, Wanaparthy, Warangal  **Tripura:** Khowai, North Tripura, Unakoti, South Tripura, Gomati  **Uttar Pradesh**:Fatehpur, Firozabad, Ghazipur, Jaunpur, Muzaffarnagar, Rampur, Amethi, Ballia, Banda, Barabanki, Budaun, Bulandshahar, Etawah, Ghaziabad, Gorakhpur, Kanpur Nagar, Kushinagar, Mirzapur  **Uttarakhand**- Almora, Udhamsingh Nagar, Dehradun, Haridwar, Rudraprayag  **West Benghal**- Cooch Behar, Howrah, Malda, Murshidabad, North 24 Parganas, Bankura, Birbhum, Bishnupur, Nadia, Paschim Medinipur, Rampurhat, Behala, Jalpaiguri, Maniktala, MTMTB  **Rajasthan-** Baran, Bhilwara, Chittaurgarg, Dholpur, Jaipur I, Jaipur II, Jaisalmer, Jhunjhunun, Sawai, Madhopur, Sikar, Udaipur, Alwar, BAnswara, Bundi, Churu, Dungarpur, Hanumangarh, Karauli, Tonk  **Haryana**- Bhiwani, Faridabad, Gurgaon, Jhajjar, Kaithal, Rewari, Rohtak, Yamunanagar, Ambala, Kurukshetra, Mewat, Panchkula, mahendragarh  **Goa**- North & South Goa  **Jammu & Kashmir:** Badgam, Baramula, Jammu, Poonch, Rajouri, Anantnag, Doda, Kathua, Kupwara, Pulwama, Srinagar, Udhampur  **Himachal**- Hamirpur HP, Mandi, Una-HP  **Jharkhand:** Palamu, Saraikela  **Lakshadweep**: Lakshadweep  **Karnataka**: Bangalore city, Bangalore urban, Chikkaballapur, Dakshina Kannada, Haveri, Shimoga, Kalaburagi, Mandya, Belgaum  **Gujarat:** Ahmedabad Rural, Banaskantha, Batod, Chhotaudepur, Jamnagar, Junagadh, Kachchh, Morbi, Navsari, Patan, Porbandhar, Rajkot, Surat MC,Surendranagar, The Dangs,  Vadodara Municipal Corporation, Valsad, Vyara, Amreli, Mahisagar, Bhavnagar, Gir Somnath, Panchmahals, Vadodara rural  **Kerala:** Alappuzha, Ernakulam, Idukki, Kasaragod, Kollam, Kottayam, Malappuram, Pathanamthitta, Thiruvanathapuram, Thrissur, Wayanad |
|  | **List of districts with none of the TB ACF quality indicators met.**  **Andhra Pradesh-**Guntur, Kurnool  **Arunachal Pradesh:** Changlang, East Kameng, East Siang, Lohit, Papumpare, Tawang, Tirap, Upper subansiri  **Assam:** Goalpara, Kokrajhar, Tinsukia, Udalguri  **Bihar:** Bhojpur, Purnia  **Chattisgarh:** Bemetara, Dhamtari, Gariyabund, Janjgir-champa, Narayanpur, Raipur, Surajpur  **Delhi:** CD chest clinic, Chest clinic Narela, Kingsway  **Madhya Pradesh:** Chhindwara, Dewas, Khandwa, Sehore, Ujjain  **Maharashtra:** Ahmednagar MC, Andheri West, Kalayam Dombivli MC  **Manipur**- Churachandpur, Senapati  **Meghalaya**: West Garo Hills, West Khasi Hills  **Mizoram**: Lawngtlai  **Nagaland:** Dimapur, Zunheboto  **Odisha:** Balangir, Sundargarh  **Punjab**: Faridkot, Mansa-PN  **Sikkim**- South District  **Tamil Nadu**- Coimbatore, Dharmapuri, Kanniyakumari, Namakkal, Pudukkottai, Salem, Sivagangai, South Chennai, Tiruppur, West Chennai  **Telangana:** Hanumakonda, Kamareddy, Nizamabad  **Tripura**: Sephahijala, West Tripura  **Uttarpradesh:** Basti, Kanshiram Nagar, Pilibhit, Prayagraj, Varanasi  **Uttarakhand**: Bageshwar, Chamolo, Nainital, Tehri Garhwal  **West Benghal**: Alipore (Kolkata), Alipurduar, Basirhat, Dakshin Dinajpur, Darjiling, Diamond Harbour, Hazi (Kolkata), Hoogly, Jhargram, Kalimpong, Nandigram HD, Paschim Bardhaman, Purba Bardhaman, Purba Medinipur, Purulia, South 24 Parganas, Tollygunge (Kolkata)  **Rajasthan:** Ajmer, Barmer, Bharatpur, Bikaner, Dausa, Jalore, Jodhpur, Kota, Nagaur, Pali, Rajsamand  **Haryana:** Charkhi Dadri, Fatehabad, Hisar, Jind, Karnal, Palwal, Sirsa, Sonipat  **Ladakh:** Leh, Kargil  **Jharkhand**: Koderma  **Karnataka**: Bellary, Chamarajanagar, Chitradurga, Hassan, Ramanagara, Tumkur, Uttara Kannada, Yadgiri, Kodagu  **Gujarat:** Gandhinagar, Kheda, Mahesana  **Kerala:** Kannur, Kozhikode |
| **2** | **Districts that met the % population screened and NNS cut-offs** |
|  | **List of districts with NNS met (</= 1538) and % Screened is >/=10%**  **Uttar Pradesh:** Ayodhya, Etah, Gonda, Shahjahanpur  **Nagaland:** Longleng  **Madhya Pradesh**: Balaghat, Hoshangabad, Seoni, Shajapur  **Manipur:** Chandel  **Maharashtra:** Akola Mc, Bhandara, Dhule MC |
|  | **List of districts with NNS met (</= 1538) and % Screened is >/=5%**  **Andhra Pradesh**: Kurnool  **Bihar:** Sheikhpura  **Arunachal Pradesh:** Changlang, East Siang, Tirap  **Uttarakhand:** Haridwar  **Telangana:** Jayashankar Bhupalpally, Kumurambheem Asifabad, Wanaparthy  **Uttar Pradesh**: Ayodhya, Etah, Gonda, Shahjahanpur, Ballia, Banda  **Rajasthan:** Banswara  **Nagaland:** Longleng, Zunheboto  **Madhya Pradesh**: Balaghat, Chhindwara, Guna, Hoshangabad, Khargone, Seoni, Shajapur, Umaria  **Manipur:** Chandel, Senapati, Tamenglong, Ukhrul  **Maharashtra:** Akola MC, Bhandara, Bhiwandi Nizampur, Dhule MC  **Haryana:** Mewat, Sirsa |
|  | **List of districts with NNS met (</= 769) and % Screened is >/=10%**  **Madhya Pradesh:** Balaghat, Shajapur |
|  | **List of districts with NNS met (</= 769) and % Screened is >/=5%**  **Bihar:** Sheikhpura,  **Telangana:** Jayashankar Bhupalpally, Kumurambheem Asifabad, Wanaparthy  **Madhya Pradesh:** Balaghat, Shajapur, Umaria  **Maharashtra:** Bhiwandi Nizampur |

TB-tuberculosis; ACF- active case finding; NNS: number needed to screen
